# Measurement of CT radiation beam width with a pencil ionization chamber and radiopaque mask

**DOI:** 10.1002/acm2.70027

**Published:** 2025-02-27

**Authors:** Rani Al‐Senan, David M. Gauntt, Gary T. Barnes

**Affiliations:** ^1^ Penn State University Hershey Pennsylvania USA; ^2^ University of Alabama at Birmingham Medical Center Birmingham Alabama USA

**Keywords:** ACR, beam width, CT, pencil ion chamber

## Abstract

**Purpose:**

This study compares fan beam CT scanner radiation beam widths measured with a pencil ionization chamber‐radiopaque mask technique with commonly used film and computed radiography (CR) measurements.

**Methods:**

For a given fan beam CT scanner x‐ray beam, the ionization chamber‐mask technique determines the radiation beam width by exposing a 100 mm pencil chamber with and without a radiopaque cylindrical mask of known width that is a fraction of the nominal beam width. Additional widths can then be measured using the same kV, mAs, and pre‐patient filtration without the mask. CT scanner radiation beam width measurements with the technique were compared with film and CR techniques.

**Results:**

Measurements from 20 different detector configurations/focal spot combinations on fan beam CT scanners from two manufacturers are presented. The root mean square (RMS) difference between the ionization chamber‐mask measured beam widths and film measurements was 0.31 mm, with a similar RMS difference of 0.28 mm with CR measurements. These results compare favorably with the RMS difference between film and CR measurements, which was 0.35 mm.

**Conclusion:**

This study demonstrates that radiation beam widths of fan beam CT scanners can be measured using the ionization chamber‐radiopaque mask method with an RMS accuracy of better than 0.5 mm. We demonstrate the method is applicable to nominal beam widths ranging from 1 to 40 mm.

## 1 INTRODUCTION

The American College of Radiology (ACR), in their CT Quality Control Manual, recommends the routine measurement of radiation beam width during annual quality assurance (QA) surveys of CT machines.^1^ This recommendation has been reinforced by the ACR mandating that for a CT site to be accredited, the site must have a documented CT quality control program consistent with the recommendations of the 2017 CT Quality Control Manual.^2^ Similarly, the American Association of Physicists in Medicine (AAPM) Report Number 233 also recommends annual measurement of CT beam width for every unique collimation setting.^3^

Measuring CT scanner x‐ray beam widths presents challenges, as the beam width can change with detector configuration and focal spot size, requiring several measurements. Various techniques have been devised for measuring CT x‐ray beam width, using thermoluminescent dosimeters (TLDs),^4^ self‐developing radiosensitive film, computed radiography (CR) imaging plates, optically stimulated luminescence (OSL) dosimeters,^3^ and solid‐state pencil radiation profile meters.^5^ These techniques are time consuming, expensive, or both.

Radiochromic film, though widely used, demands higher exposures for noticeable darkening. After exposure, this film can be read manually or digitized for analysis. However, the latter requires specialized tools or meticulous analysis to achieve high accuracy. When employing CR plates, precautions must be taken to prevent overexposure, which can lead to saturation and distortion of the profile.^6^ The use of digital calipers or spreadsheet programs with linearized images facilitates the analysis of CR plate images.

At UAB (University of Alabama Birmingham), a technique was developed to measure CT radiation beam widths utilizing a CT pencil ionization chamber coupled with a radiopaque mask. This approach offers a practical and time‐efficient solution to the time commitment and challenges associated with TL and OSL dosimeters, radiochromic film, and CR measurement techniques.^7^

## 2 MATERIALS AND METHODS

**FIGURE 1:**
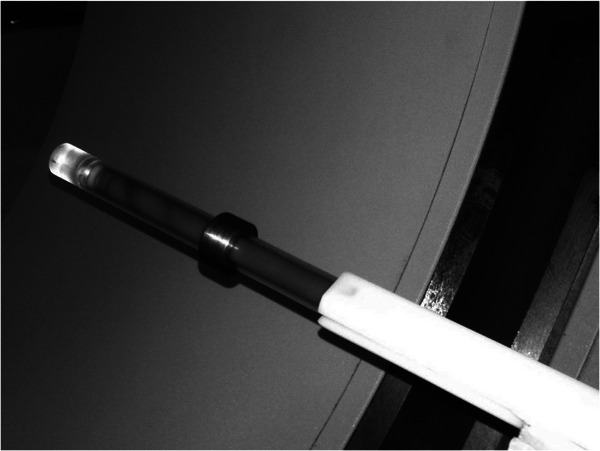
Pencil ionization chamber with tungsten mask positioned CT scanner gantry.

A 100 mm pencil ionization chamber is placed along the z‐axis of the scanner, with the center of the sensitive region located at isocenter (i.e., *z* = 0). The detector coverage should be set to the largest possible value, that is, 80 mm or smaller. A hollow radiopaque cylindrical mask with an effective length dimension, ***L_eff_***, (a factor of two or so smaller than the nominal radiation beam width being measured and thick enough to completely attenuate a CT beam) is positioned on the chamber and centered on z = 0 as shown in Figure 1. A scan is taken and the exposure with the mask is measured and recorded. The mask is removed and a second scan with identical parameters is acquired, with the exposure measured and recorded. The difference of the two exposures, $X_{w/o\;mask}-X_{w/mask},$ is the exposure associated with an x‐ray beam attenuated by the mask, so the exposure at isocenter per millimeter (the mask calibration) is:

(1) $$X/L=\frac{X_{w/o\;mask}-X_{w/mask}}{L_{eff}}\;$$

where ***X_w/ mask_*** and ***X_w/o mask_*** are the exposures with and without the mask. The radiation beam width, ***W_N_*** *_x_* ***_T,_*** of the exposure for nominal collimation *N x T* without the mask is given by

(2) $$W_{N\times T}=\frac{X_{N\times T}}{X/L}\;$$

where ***X_N_*** *_x_* ***_T_*** is the measured exposure for a any nominal beam width *N x T* and the same scan parameters (kV, mA, exposure time, focal spot size, and pre‐patient filtration/scan FOV) as the conditions used to determine *X/L*. Once the mask calibration, *X/L*, has been determined, the measured beam widths for exposures without the mask and the same scan parameters but for other nominal beam widths is given by Equation (2).

**FIGURE 2:**
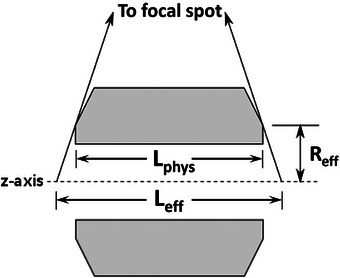
Cross‐sectional view of the mask showing the definition of ***R_eff_***, ***L_phys_***, and ***L_eff_***. Angles of the x‐rays and chamfer are exaggerated for illustrative purposes.

The effective length of the mask is determined by various factors, including its physical length, magnification onto the isocenter, and x‐ray transmission through the mask. Shown in Figure 2 is a cross‐section of the mask design employed illustrating its effective length, ***L_eff_*,** and the projected effective radius, ***R_eff_*** (the distance from the central axis of the mask to the edge of the mask that defines the end of its radiographic shadow). For a perfectly radiopaque mask, ***L_eff_*** would be the shadow of the mask projected onto the z‐axis passing through the isocenter.

(3) $$L_{eff}=L_{phys}\;\times \;M_{geom}=L_{phys}\;\frac{SID}{SID-R_{eff}}$$

where ***L_phy_*** is the mask's physical length, *M_geom_* is the geometric magnification, and ***SID*** is the source‐to‐isocenter distance. The small chamfer (or beveled edge) of the mask has two purposes: it reduces ***R_eff_*** reducing the mask's magnification and effective length, and reduces the sensitivity of the effective length to tilt positioning errors.

The ion chamber method was evaluated on two CT scanners located at the University of Alabama Medical Center. The CT scanners, nominal beam widths and associated detector configurations are listed in Table 1. Data collection was standardized at 80 kV, representing the lowest x‐ray tube voltage setting on each scanner. The low kV minimized the effects of collimator‐induced leakage and mask wall transmission.

**TABLE 1:** CT scanners and associated nominal beam widths measured.

Manufacturer and model	Philips Brilliance 40	GE Discovery 750 HD
Detector configurations and nominal beam widths	2 × 0.5 = 1 mm 2 × 0.625 = 1.25 mm 12 × 0.625 = 7.5 mm 16 × 0.625 = 10 mm 20 × 0.625 = 12.5 mm 12 × 1.25 = 15 mm 40 × 0.625 = 25 mm 32 × 1.25 = 40 mm 16 × 2.5 = 40 mm	1 × 1.25 = 1.25 mm 2 × 1.25 = 2.5 mm 4 × 1.25 = 5.0 mm 16 × 0.625 = 10 mm 32 × 0.625 = 20 mm 64 × 0.625 = 40 mm
Source‐isocenter distance	570 mm	539 mm
***L_eff_***	10.475 mm	10.483 mm

The General Electric scanner displayed “Dose Efficiency” on the console; this is the nominal beam width divided by the actual collimated width. For any given detector configuration, two dose efficiencies are displayed, one at low tube currents and a lower efficiency at high tube currents. These correspond to the two different focal spot sizes on the scanner. Measurements were made at both high and low tube currents.

**FIGURE 3:**
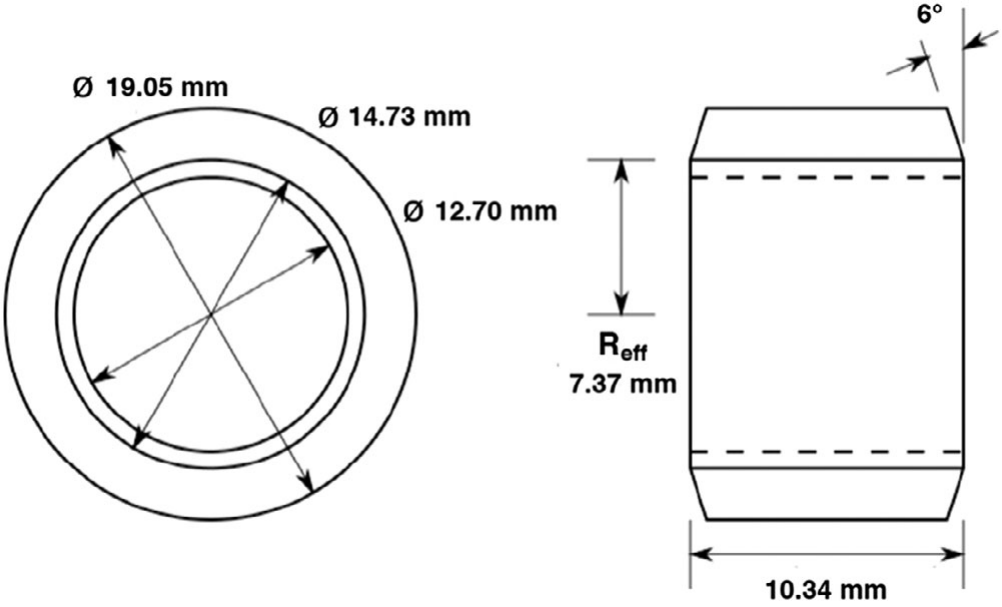
Mechanical drawing of tungsten mask. In the drawing, the chamfer is greater than 6° for illustrative purposes. The chamfer has the effect of reducing magnification of the mask while maintaining wall thickness and wall x‐ray absorption efficiency.

Figure 3 is a mechanical drawing of the mask. The mask was fabricated at the UAB Physics Department machine shop and consists of a cylinder 19.05 mm in diameter and 10.34 mm long, with a 12.7 mm diameter hole drilled along the axis, and is made of tungsten alloy MT‐17C (90% tungsten, 6% nickel, and 4% copper). The tungsten alloy was chosen in preference to lead because it has a lower x‐ray transmission and is more mechanically robust. The outside diameter is that of the smallest diameter rod normally stocked by Midwest Tungsten Service (Willowbrook, Illinois, USA), which would allow at least a 3 mm wall thickness. The effective radius is 7.37 mm and the mask wall is 3.17 mm thick.

**FIGURE 4:**
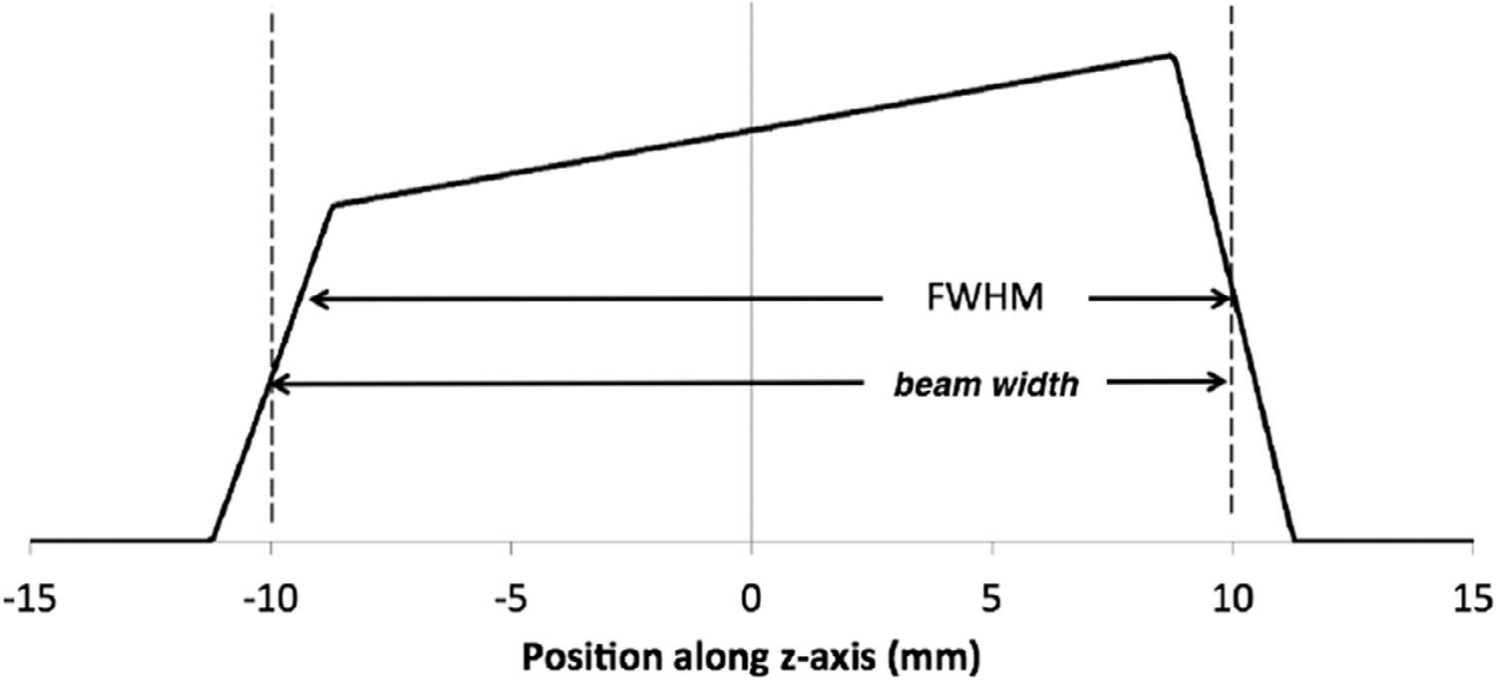
CT radiation profiles along the z‐axis have an asymmetry associated with the heel effect. “Beam width” is the the radiation beam width measured with film and CR: the distance from 1/2 the height of the line fit to the rising edge to 1/2 the height of the line fit to the falling edge. For comparison, the FWHM (full‐width‐half‐maximum) of the profile is shown.

For comparison, beam widths were measured using film and CR cassette techniques. In each case, the image receptor (either radiochromic film or a CR cassette) was exposed in an axial scan. Subsequently, the recorded image was converted to a radiation dose profile with the pixel value linearly related to the exposure at the image receptor. The profile was fitted with three lines as shown in Figure 4: line 1 to the rising edge; line 2 to the plateau; and line 3 to the falling edge. The beam widths reported are the distances from the 1/2 the height of the rising edge to 1/2 the height of the falling edge of the radiation dose profiles.

To measure the beam width using radiochromic film, pieces of Gafchromic film (XR‐CT3, Ashland, Bridgewater, New Jersey, USA) were cut into strips approximately 1 cm wide and 10 cm long. The pencil chamber was positioned at isocenter, extending past the end of the patient table. One strip was placed on the pencil chamber centered on the central image plane and an axial scan was made. Upon acquiring all film strips for a single scanner, the exposed strips along with one unexposed strip were digitized at a resolution of 12 pixels per mm employing a reflective scanner (Officejet 5610v All‐in‐One, HP, Palo Alto, California, USA) and profiles from the resultant film density scans converted to dose profiles using established techniques^8^ and the beam widths were determined as described above.

For the CR beam width measurements, 20 cm × 25 cm CR orthopedic cassettes (Fuji Type C IP cassette, FujiFilm, Tokyo, Japan) were employed. The cassette was placed on the scanner table with the long axis parallel to the scanner z‐axis, centered on the central image plane, and table height adjusted so the cassette was at isocenter. A single axial scan was acquired for each nominal beam width and detector configuration studied. A low technique (80 kV, 10 mAs/rotation) was used to avoid saturating the cassette. No CR measurements were taken for the high current (large focal spot) data sets for the GE scanner as the radiation level would have saturated the CR cassettes. Orthopedic cassettes were employed because of their smaller image pixel pitch (100 µm) compared to the 200 µm pitch of Fuji general‐purpose cassettes. Following exposure, the CR cassette was read [Fuji CR reader (FCR 5000 Plus, Fujifilm Medical Systems, Tokyo, Japan]. The “For Processing” image was extracted and the beam widths determined.

Additionally, the average difference, the RMS difference, and the maximum difference in the beam width measurements were calculated for each pair of methodologies (film—CR, ion chamber—CR, and ion chamber—film).

## 3 RESULTS

Table 2 presents the beam width measured with the three techniques employing the small focal spot on the GE scanner along with the associated nominal beams, detector configurations *(N x T)*, dose efficiencies, and calculated beam widths. Table 3 presents the beam width measured with the film and ion chamber techniques employing the large focal spot on the GE scanner along with the associated nominal beams, detector configurations *(N x T)*, dose efficiencies, and calculated beam widths. Table 4 presents the beam width measured with the three techniques on the Philips scanner along with the associated nominal beams, and detector configurations *(N x T)*. The dose efficiencies and calculated beam widths are not listed in Table 4, as the dose efficiency was not displayed on the Philips scanner console. Presented in Table 5 are the average difference, average RMS difference, and the maximum difference between the comparison film and CR, ion chamber and CR, and ion chamber and film beam width measurements.

**TABLE 2:** Measured beam widths for GE Discovery 750 HD, small focal spot (200 mA, 1 s/rot).

				Measured beam width (mm)		
Nominal beam width and detector configuration		Nominal dose efficiency	Calculated beam width (mm)	Film	CR	Ion chamber
40 mm	64 × 0.625	92.6%	43.20	42.67	42.90	43.18
20 mm	32 × 0.625	87.4%	22.88	22.24	22.30	22.65
10 mm	16 × 0.625	77.0%	12.99	12.38	12.55	12.80
5 mm	4 × 1.25	63.9%	7.82	7.13	7.40	7.53
2.5 mm	2 × 1.25	56.4%	4.43	4.04	4.23	4.32
1.25 mm	1 × 1.25	41.5%	3.01	2.72	2.78	2.79

**TABLE 3:** Measured beam widths for GE Discovery 750 HD, large focal spot (700 mA, 1 s/rot).

				Measured beam width (mm)		
Nominal beam width and detector configuration		Nominal dose efficiency	Calculated beam width (mm)	Film	CR	Ion chamber
40 mm	64 × 0.625	91.9%	43.53	43.65	N/A	43.54
20 mm	32 × 0.625	85.4%	23.42	22.08	N/A	23.03
10 mm	16 × 0.625	72.7%	13.76	13.26	N/A	13.40
5 mm	4 × 1.25	57.9%	8.64	8.34	N/A	8.51
2.5 mm	2 × 1.25	53.1%	4.71	4.04	N/A	4.13

The CR cassette method cannot be used at this tube current because the cassette would saturate.

**TABLE 4:** Measured beam widths for Philips Brilliance 40 (400 mA, 1 s/rot).

				Measured beam width (mm)		
Nominal beam width and detector configuration		Nominal dose efficiency	Calculated beam width (mm)	Film	CR	Ion chamber
40 mm	16 × 2.5	N/A	N/A	41.41	41.89	41.39
40 mm	32 × 1.25	N/A	N/A	42.70	43.00	42.24
25 mm	40 × 0.625	N/A	N/A	30.83	31.71	31.28
15 mm	12 × 1.25	N/A	N/A	18.95	19.45	19.07
12.5 mm	20 × 0.625	N/A	N/A	17.59	18.04	18.30
10 mm	16 × 0.625	N/A	N/A	15.68	15.29	15.60
7.5 mm	12 × 0.625	N/A	N/A	12.27	11.76	12.19
1.25 mm	2 × 0.625	N/A	N/A	5.00	4.70	4.77
1 mm	2 × 0.5	N/A	N/A	4.38	3.97	3.98

The nominal dose efficiency is not displayed on the console of this scanner.

**TABLE 5:** Summary of the differences between pairs of the three different beam width measurement methods studied.

Parameter	Film—CR	Ion chamber—CR	Ion chamber—Film
Average difference (mm)	0.13	0.01	0.17
Average RMS difference (mm)	0.35	0.28	0.31
Maximum difference (mm)	0.88	0.76	0.95

## 4 DISCUSSION

Noteworthy is the good agreement between the three beam width measurement methodologies. The maximum differences listed in Table 5 for the three comparisons were comparable and less than 1.0 mm, the average differences were small (< 0.2 mm) as one would expect from a sampling of random differences; and the average RMS differences were comparable and less than 0.5 mm. Our results are consistent with film and CR comparison results of Jackson et al., who reported an RMS difference of 0.72 mm and a maximum difference of 0.9 mm for four nominal beam widths.^7^

In practice, we calibrate the mask, ***X/L***, for the largest nominal beam width on a scanner and then expose the pencil ion chamber with the same technical factors (kV, mA, exposure time, and pre‐patient filtration/scan FOV) for the other nominal beams widths, record the readings, and employ Equation (2) to determine the associated measured beam widths. For each reading, three exposures are made to confirm the exposure reproducibility of the unit and the three measurements are averaged.

**FIGURE 5:**
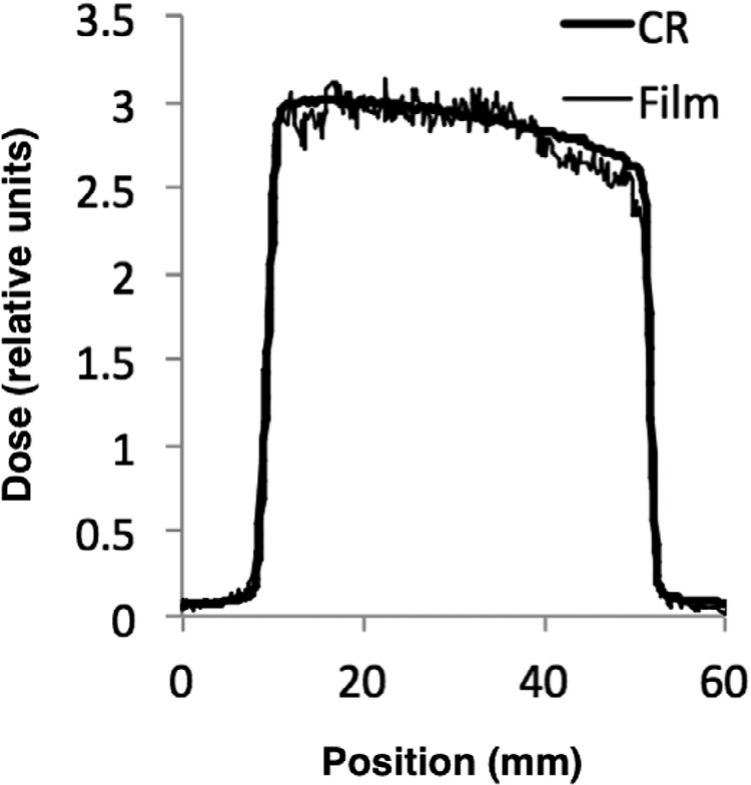
Measured CR and film radiation dose profiles for GE CT scanner. 40 mm nominal beam width (64 × 0.625 detector configuration).

Shown in Figure 5 are film and CR radiation dose profiles obtained for a 40 mm nominal beam width on the GE scanner. The heel effect results in a plateau slope of ≈ 0.33%/mm. The mask calibration estimates the average around center of the mask. There are three potential sources of error associated with the ion chamber CT radiation beam width measurement we considered: (1) the mask and ion chamber are not at isocenter; and (2) the ion chamber axis is tilted relative to the z‐axis of the reconstructed slices. In practice, the first error can be minimized by viewing the central reconstructed image and confirming that the mask and ion chamber are at the center of rotation. Likewise, the second error can be minimized by viewing the central reconstructed images and confirming the mask is centered on z = 0. If the mask is positioned at z = ± 1.0 mm rather than at z = 0, for a heel effect slope of 0.33%/mm, the error in the mask calibration would be ≈ 0.5% (or about 0.2 mm for a 40 mm nominal width).

The ion chamber tilt error can be evaluated by comparing the location of the ion chamber in the first and last slices of an acquisition. If the position of the ion chamber does not shift between the first and last slice it is not tilted. For a 20 mm nominal beam width, a 5° tilt would be apparent as it would result in a 1.75 mm shift in the location of the center of the ion chamber between the first and last slices and a 3.5 mm shift for a 40 mm nominal beam width. A smaller tilt would be less obvious. With the tungsten mask described above, our measurements indicate that a 2.5° tilt would result in a mask calibration error of less than 1%. The error would be greater if the mask did not have a chamfer.

The chamfer of the mask used in our experiments was 6° (Figure 3), and the mask was designed before we made our measurements; the design was based in part on minimizing machine shop requirements and on the tungsten rod diameters commercially available. The chamfer reduced the magnification of the mask's physical length by a small amount (i.e., from ≈1.02 to ≈1.01) but reduced the minimum wall thickness to ≈ 1 mm, which is still highly attenuating and reduces sensitivity to misalignment errors. A 10 mm (physical length) mask combined with a typical (540–570 mm) CT scanner source‐to‐isocenter distance results in a cone angle of ≈ 0.5°. With a chamfer of 0.5°, the system is sensitive to misalignments, but with a chamfer of 6°, the sensitivity is reduced for misalignments of up to ≈ 5°. Based on our experience, the pencil chamber (and mask) can be aligned with the isocenter axis to less than 1.5°. Assuming that the misalignment of the pencil chamber is < 1.5°, the 6° chamfer is larger than needed and a chamfer of 2°–3° is adequate.

The widest beam studied had a nominal width of 40 mm. The technique will likely not be useful for scanners with beam widths larger than ≈ 80 mm simply because the standard pencil chamber is 100 mm long. Also, the results reported above are for axial scans with the ion pencil chamber stationary and supported by scanner's patient couch. It should also be noted that the method is applicable to helical scans provided the ion chamber is supported on the scanner's gantry shroud and held stationary as described by DePew et al.^9^

Off‐focus radiation contributes to the radiation output. If there were significant off‐focus contributions in the CT scanners studied, determining the radiation beam width from film and CR radiation profiles as we did would be less sensitive to the off‐focus contributions than would the ion chamber technique. The good agreement we observed between the ion chamber and the film and CR beam width measurements is confirmation that off‐focus radiation is minimal in the CT scanners studied.

RaySafe (Unfors RaySafe AB, Sweden) recently published Application Note 2024‐04‐09 “Measuring CT beam width with RaySafe X2.”^10^ The note describes two methods for measuring CT beam width using the RaySafe X2 R/F sensor and the 100‐mm pencil ion chamber. The first requires that the x‐ray tube and scanner table be stationary (not routinely available to the clinical medical physicist), and the second RaySafe method uses a stationary table but an axial scan. Using two detectors makes the Raysafe methods susceptible to calibration errors. Additionally required are tools (not provided) for positioning the two different sensors at isocenter. We have made a limited number of beam width measurements comparing the RafeSafe axial method with our method. The results differed by < 3%. In our experience, the RafeSafe approach takes more time due to two factors: (1) positioning two sensors (with different calibrations) rather than one at isocenter; and (2) the time required to use the RafeSafe dose rate graphical capability to determine the maximum axial scan isocenter dose rate. Of interest for future work is an in‐depth comparison of the ion chamber and mask, RafeSafe, and radiochromic film CT beam width measurements.

## 5 CONCLUSION

We have demonstrated that it is possible to measure the radiation beam width of a CT scanner using a pencil ionization chamber and radiopaque mask, with an RMS accuracy of better than 0.5 mm. The only equipment required that is not part of the typical CT medical physics inventory is a small tungsten mask, which we were able to manufacture for < $100. Taking the precautions noted above, mask positioning errors were minimized and did not contribute any significant errors to the results. The ACR CT QC Manual indicates, when acceptance testing a CT scanner or performing an annual audit, that every clinically used beam width be measured.^1^ The time requirements for ion chamber measurement of several beam widths are minimal, and it should be possible to measure the 6–10 available beam widths on a typical CT scanner in less than 5 min.

## CONFLICT OF INTEREST STATEMENT

UAB has an agreement with Radcal (Monrovia CA 91016) to market the pencil ion chamber mask design.
